# Comparing Artificial Intelligence Large Language Models in Medical Training: A Performance Analysis of ChatGPT and DeepSeek on United States Medical Licensing Examination (USMLE) Style Questions

**DOI:** 10.7759/cureus.90212

**Published:** 2025-08-16

**Authors:** Runze Zhang, Qinyun Cai, Amanda Sartori, Nasser Gayed, Heather Collette

**Affiliations:** 1 Department of Medicine, College of Medicine, University of Illinois at Chicago, Chicago, USA; 2 Department of Medicine, Carle Illinois College of Medicine, Urbana, USA; 3 Department of Medicine, Chicago Medical School, Rosalind Franklin University of Medicine and Science, North Chicago, USA; 4 Department of Biomedical and Translational Science, Carle Illinois College of Medicine, Urbana, USA; 5 Department of Pediatrics, Yale School of Medicine, New Haven, USA

**Keywords:** artificial intelligence in education, artificial intelligence in medicine, innovation in medical education, medical education technology, medical school education, united states medical licensing examination (usmle), usmle step 1, usmle step 2 ck

## Abstract

Introduction

The integration of artificial intelligence (AI) into medical education is reshaping how students prepare for standardized examinations. Prior studies have shown that AI models can achieve high accuracy on United States Medical Licensing Examination (USMLE) questions, highlighting their potential for examination preparation. ChatGPT (GPT), especially the 4o model, is one of the most widely used AI models; however, its accessibility is limited by subscription costs and regional censorship. DeepSeek (DS), a newer AI model, offers free access and has demonstrated comparable performance in general tasks. In this study, we compared the performance of GPT-4o and DS DeepThink R1 on the AMBOSS medical board preparation question bank to evaluate their potential and limitations as supplementary tools in medical education.

Methods

We extracted 1,079 USMLE-style multiple-choice questions from the AMBOSS question bank. Questions were categorized by USMLE Step 1 and Step 2 examinations and further grouped by topic, resulting in 36 categories. Each question was assigned a difficulty level (easy, intermediate, or hard) based on AMBOSS grading criteria. To ensure balanced representation, we randomly selected 10 questions per difficulty level per category. Questions and answer choices were copied verbatim from the AMBOSS website and input into GPT-4o and DS R1 without any modification. Model responses were scored as correct or incorrect, and correctness rates were compared across GPT-4o, DS R1, and AMBOSS user performance.

Results

Both GPT and DS outperformed AMBOSS users, with overall accuracies of 88.79%, 78.68%, and 56.98%, respectively. Comparing GPT and DS, GPT performed significantly better overall (t=7.90, p<0.0001). When stratified by examination type, GPT achieved significantly higher accuracy than DS in both Step 1 (0.89 vs. 0.78, p < 0.0001) and Step 2 (0.88 vs. 0.80, p < 0.0001). GPT consistently showed higher accuracy than DS at all three difficulty levels. However, when further stratified by examination type, statistically significances were only observed in intermediate (p = 0.0002) and hard (p = 0.0021) questions in both Step 1 and Step 2.

Conclusion

Our findings demonstrated that both AI models outperformed human learners, with GPT-4o showing superior accuracy, particularly in intermediate and hard questions. While DS underperformed relative to GPT, its free accessibility and competitive accuracy in easy questions suggest that it may serve as a viable alternative, particularly in resource-limited settings.

## Introduction

The integration of artificial intelligence (AI) in medical education is reshaping how medical students learn and prepare for standardized examinations. Large language models (LLMs) such as ChatGPT (GPT) and DeepSeek (DS) are increasingly being used as study aids by providing simplified explanations, clinical correlations, and diagnostic reasoning [1]. These models offer students an accessible and seemingly credible resource, reducing reliance on textbooks and institution-provided material. Recent studies have evaluated AI’s ability to perform on standardized medical question banks, such as AMBOSS and USMLE-style examinations, offering insight into its accuracy across different difficulty levels and medical systems [2-3]. Since its introduction, AI models such as GPT have demonstrated remarkable performance on various medical board examinations. For instance, GPT achieved scores at or near the passing threshold on the United States Medical Licensing Examination (USMLE) Steps 1, 2CK, and 3 without specialized training, suggesting potential applications in medical education and clinical decision-making [4]. Further studies have shown that GPT-4o, an advanced version of GPT, has achieved even higher accuracy rates on USMLE-style questions, indicating rapid advancements in AI capabilities within medical contexts [3]. However, while AI can enhance learning efficiency in some capacity, it also introduces risks, as students may develop overreliance on AI-generated responses without critically evaluating their accuracy, especially when models provide incorrect yet confident explanations [5]. Therefore, guiding students in selecting more accurate and reliable models is essential to minimize misinformation and promote responsible integration into study routines.

Medical students increasingly rely on AI-powered platforms to answer clinical questions, summarize medical literature, and clarify complex concepts [6]. While LLMs achieved respectable accuracy on USMLE-style questions, they are not always reliable, as they can generate inaccurate, outdated, or misleading information [2]. This issue is compounded by AI’s tendency to produce responses that sound plausible but may be incorrect - an occurrence known as "hallucination." Additionally, the lack of transparency in AI model outputs raises concerns, as students may accept AI-generated explanations without questioning their accuracy or verifying sources, potentially using or propagating false information [7]. Unlike human instructors, who can provide evidence-based reasoning, AI models generate responses based on prior training and accessible databases. These limitations highlight the need for ongoing systematic evaluation of AI’s role in medical education, ensuring that students develop strong critical thinking skills while integrating AI tools responsibly in a rapidly changing intellectual environment.

DS R1 is a newer LLM released in January 2025 with a focus on advanced reasoning tasks, making it an attractive option for medical students looking to supplement their education. Thus far, DS has been proved comparable to the more established GPT-4o for many uses. However, DS’s effectiveness in medical education has not yet been published. While several studies have assessed GPT-4o’s performance in medical education, either many have been limited by the small question pools or uneven distributions of question difficulty have failed to include an equal distribution of question difficulty, thus limiting interpretations that can be made regarding performance accuracy at different difficulty levels [8-9]. Comparing the medical education performance of DS to GPT-4o, the current benchmark provides important information for students who are deciding between the two LLMs or have limited access to GPT due to the cost of a paid subscription or denied access due to censorship in countries outside the United States, such as China and Iran. This study aims to evaluate and compare the performance of GPT-4o and DS DeepThink R1 using the AMBOSS medical board preparation question bank, comparing its accuracy across different medical topics and difficulty levels against human performance. We aim to investigate the potential and limitations of AI as a supplementary tool in medical education. By analyzing AI’s capabilities in medical knowledge assessment, this study will contribute to the broader discussion on AI’s role in shaping the future of physician education and clinical decision-making.

## Materials and methods

Question selection

We randomly extracted 1,079 USMLE-style multiple-choice questions from the AMBOSS question bank (AMBOSS). AMBOSS is a widely used digital learning platform for USMLE examination preparation, offering a comprehensive question bank covering all tested topics, along with detailed explanations and performance analytics. It is commonly used by medical students for USMLE examination preparation and self-assessment [10].

Questions with images, charts, and tables were excluded from the randomization pool in this study due to limitations in DS capability, which only supports text extraction from images. We used the examination type (Step 1 or Step 2) and medical topic tags (e.g., cardiology, gastroenterology, pharmacology) provided by the AMBOSS platform to categorize the questions into 36 categories (18 per examination). Questions were classified into three difficulty levels based on AMBOSS difficulty grading criteria: easy (levels 1-2), intermediate (level 3), and hard (levels 4-5).

The distribution of questions in AMBOSS was skewed towards easy questions, with 2,554 easy (43.9%), 1,896 intermediate (32.6%), and 1,373 hard (23.6%) questions. To ensure a balanced representation for each difficulty level in the analysis, we used the AMBOSS platform’s built-in random question generator to select 10 questions per difficulty level per category, resulting in a total of 540 questions per examination. Some categories had fewer than 10 intermediate or hard questions; therefore, we supplemented with additional easy questions to maintain 30 questions per category using the same randomization process. The only exception was the Human Development category in Step 2, which contained only 29 eligible questions. For each question, we recorded the average percent correct score by human users, provided by AMBOSS. This included first-attempt data from users who spent at least 5 seconds selecting an answer.

Result collection

Questions and answer choices were copied directly from the AMBOSS website and pasted into GPT-4o (last updated: May 2024) and DS R1 (last updated: October 2023) online version without modification. Each question was input individually in LLM’s chatbox without extra commands to ensure consistent formatting across both models. For each question, we recorded a binary correctness score based on the model's response and difficulty level.

While some studies run the same question twice within the same model to ensure concordance [3], we opted to run each question only once to better simulate real-world use cases.

Statistical analysis

Data were analyzed and visualized using SAS 9.4 (SAS Institute Inc., Cary, NC) and Python 3.8.0. The two-sample t-test and paired t-test were used.

## Results

**Figure 1:**
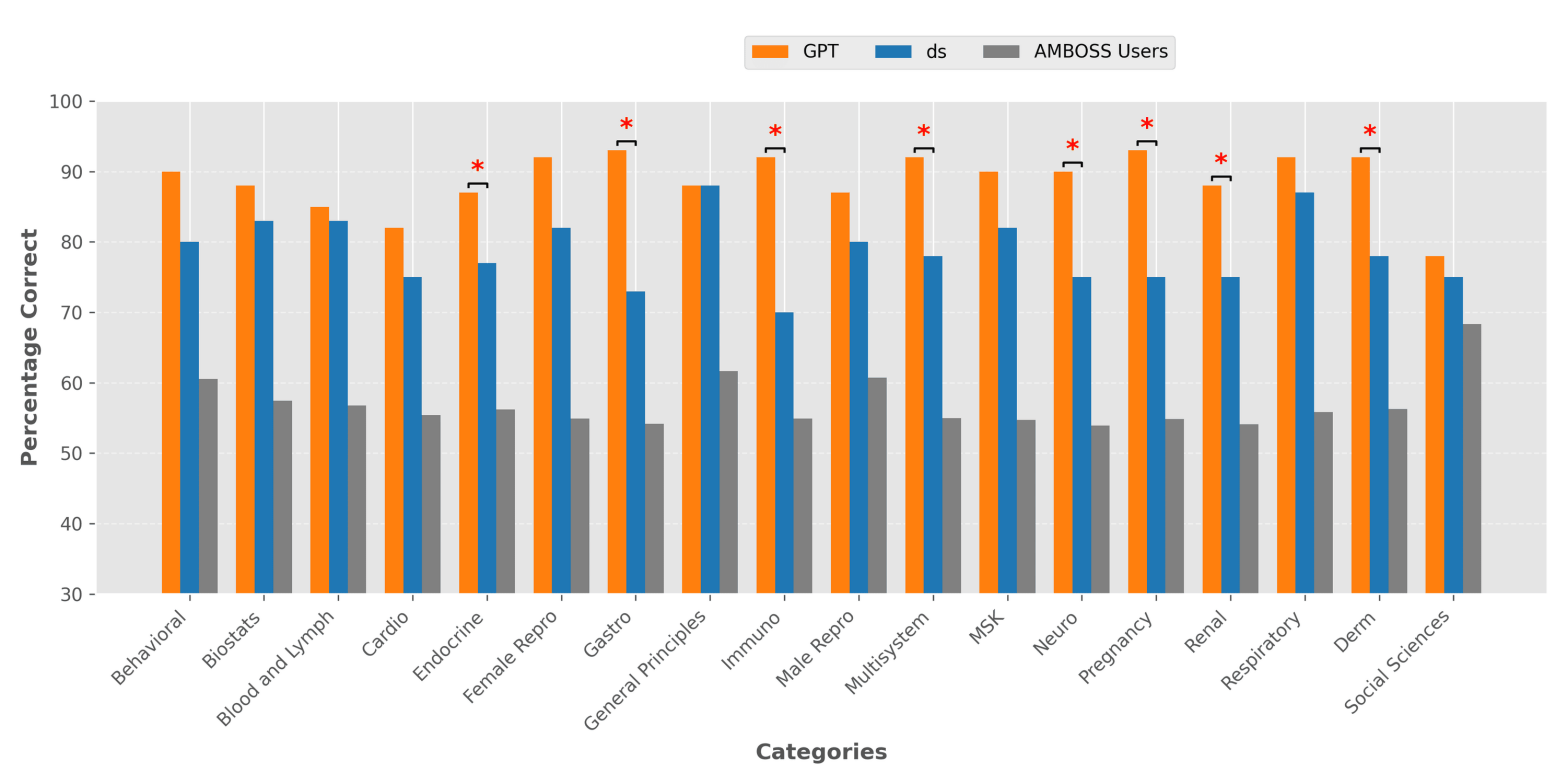
Performance comparison between GPT, DS, and AMBOSS users across AMBOSS topics on USMLE style questions. A paired t-test was performed between GPT and DS in each category. Bar graph shows the percentage of correct responses across 18 topics by three groups: GPT (orange), DS (blue), and AMBOSS users (gray). Topics are displayed along the x-axis, with the percentage of correct on the y-axis (ranging from 30% to 100%). Asterisks (*) indicate statistical significance (p < 0.05). GPT, ChatGPT; DS, DeepSeek; USMLE, United States Medical Licensing Examination

**Figure 2:**
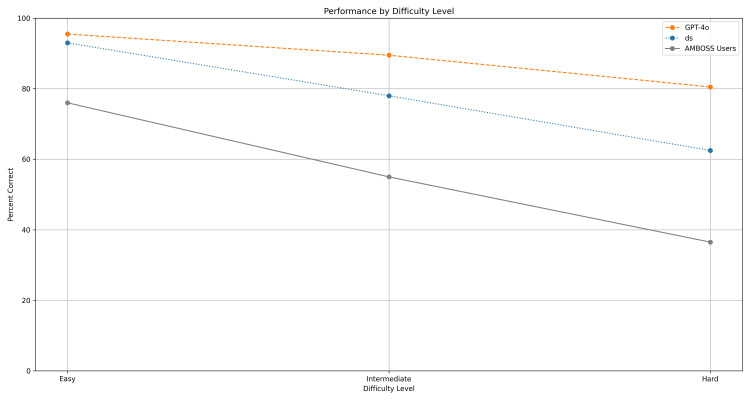
Accuracy of GPT, DS, and AMBOSS users by difficulty levels. Line graph shows the percentage of correct responses from GPT (orange), DS (blue), and AMBOSS users (gray) across easy, intermediate, and hard question categories. A Student's t-test was performed between GPT and DS at each difficulty level. Significant differences were observed at all three levels (p < 0.05). GPT, ChatGPT; DS, DeepSeek

Both GPT and DS outperformed AMBOSS users on our 1,079-question set, with accuracies of 88.79%, 78.68%, and 56.98%, respectively (Table 1, Figure 1). Comparing GPT and DS using the two-sample t-test, GPT performed significantly better overall (t=7.90, p<0.0001). When stratified by examination type, GPT achieved significantly higher accuracy than DS in both Step 1 (0.89 vs. 0.78, p < 0.0001) and Step 2 (0.88 vs. 0.80, p < 0.0001), with similar performance gaps across both examinations. When analyzed by question difficulty, GPT outperformed DS at all three difficulty levels, with the smallest accuracy difference noted for easy questions and the largest difference for hard questions (Figure 2). However, when further stratified by examination type, statistical significance was only observed in intermediate (p = 0.0002) and hard (p = 0.0021) questions for both Step 1 and Step 2.

**Table 1:** Characteristic of questions and correct rate of GPT, DS, and AMBOSS users Two-sample t-tests were used to compare the performance of GPT vs DS in each category. *P-value significance threshold is p<0.05. GPT, ChatGPT; DS, DeepSeek

Examination type	Difficulty	GPT correct	DS correct	AMBOSS users	Accuracy difference between GPT vs DS	P-value
Step 1	Easy (n=201)	96%	94%	76%	2%	0.1975
	Intermediate (n=175)	89%	76%	55%	13%	0.0002*
	Hard (n=164)	82%	60%	37%	22%	<0.0001*
	Total (n=540)	89%	78%	67%	11%	<0.0001*
Step 2	Easy (n=194)	95%	92%	76%	3%	0.0706
	Intermediate (n=177)	90%	80%	55%	10%	0.0002*
	Hard (n=168)	79%	65%	36%	14%	0.0021*
	Total (n=539)	88%	80%	57%	8%	<0.0001*
Combined	Easy (n=295)	95.5%	93%	76%	2.5%	0.0283*
	Intermediate (n=352)	89.5%	78%	55%	11.5%	<0.0001*
	Hard (n=332)	80.5%	62.5%	36.5%	18%	<0.0001*
	Total (n=1079)	88.8%	78.7%	57%	10.1%	<0.0001*

GPT demonstrated superior accuracy across nearly all topics, except for Human Development, where GPT accuracy and DS accuracy were equivalent. Statistically significant differences were noted in the topics of endocrinology (p = 0.0327), gastroenterology (p = 0.0009), immunology (p = 0.0005), multisystem disorders (p = 0.0102), neuroscience (p = 0.0188), pregnancy and childbirth (p = 0.0017), nephrology (p = 0.0313), and dermatology (p = 0.0102).

When further stratified by examination type (Step 1 vs Step 2), GPT still demonstrated superior accuracy across most topics, except for equivalent accuracy for Step 1 human development and male reproduction, and Step 2 human development. GPT showed inferior performance in Step 2 hematology (p = 0.5725) than DS. Statistically significant differences were noted in gastroenterology (p = 0.0059), immunology (p = 0.0029), multisystem disorders (p = 0.0117), neuroscience (p = 0.0169), and pregnancy and childbirth (p = 0.0014) for Step 1, as well as in nephrology (p = 0.0059) and dermatology (p = 0.0314) for Step 2.

**Figure 3:**
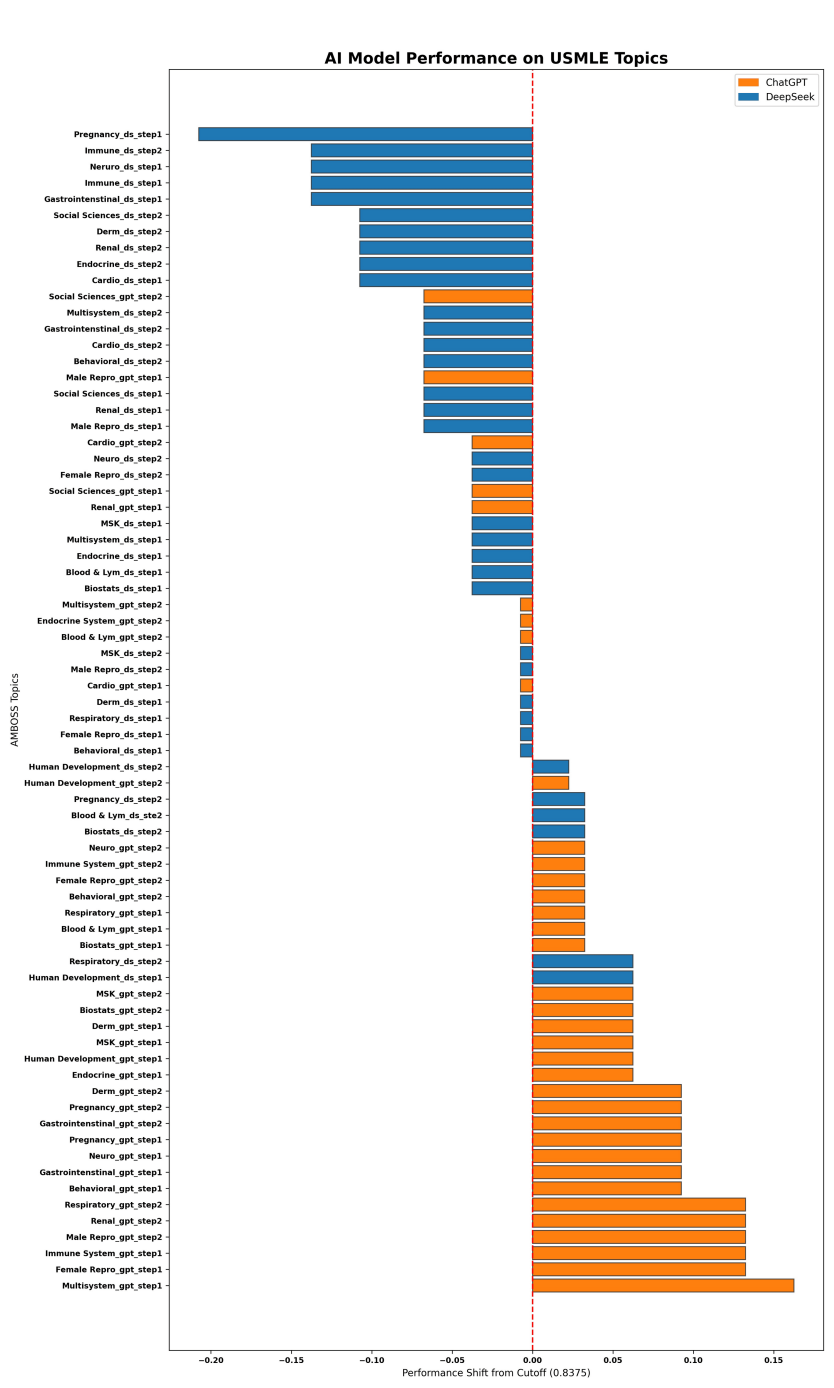
Bar graph showing performance for GPT (orange) and DS (blue) across individual AMBOSS topics for Step 1 and Step 2 questions. The red dashed line at zero indicates the overall mean accuracy across all models and topics. The x-axis represents deviation from the mean accuracy. Positive values (bars pointing right) indicate above-average performance for that topic, while negative values (bars pointing left) indicate below-average performance. AI, artificial intelligence; USMLE, United States Medical Licensing Examination; GPT, ChatGPT; DS, DeepSeek

We evaluated the accuracy of GPT and DS across 72 topic-examination type subgroups using the overall mean accuracy (0.8375) as the baseline for comparison (Figure 3). Of these subgroups, 33 performed above the baseline and 39 below it. GPT accounted for 27 of the 33 above-average subgroups, including the top 19 highest-performing ones. The six highest-accuracy subgroups were multisystem disorders (Step 1, 100%), female reproduction (Step 1, 97%), immunology (Step 1, 97%), male reproduction (Step 2, 97%), nephrology (Step 2, 97%), and respiratory system (Step 2, 97%). In contrast, DS accounted for 30 of the 39 below-average subgroups, including the 10 lowest-performing ones. The five lowest-accuracy subgroups were pregnancy and childbirth (Step 1, 63%), immunology (Step 2, 70%), neuroscience (Step 1, 70%), immunology (Step 1, 70%), and gastroenterology (Step 1, 70%).

## Discussion

Our findings demonstrate that both AI models outperformed human AMBOSS users, with GPT achieving the highest overall accuracy (88.79%) compared to DS (78.68%) and human learners (56.98%). GPT consistently outperformed DS across most question categories, with the performance gap closer for Step 2 questions than Step 1 questions. While the overall accuracy gap was similar between GPT and DS for both examinations, 5 out of 18 topics in Step 1 showed statistically significant accuracy compared to only 2 out of 18 topics in Step 2. This discrepancy is possibly because Step 1 examination questions focus on basic science and factual recall, whereas Step 2 examination questions focus on clinical reasoning and diagnosis. In addition, the greater performance discrepancy observed in Step 1 topics may have partially resulted from training data recency (May 2024 for GPT vs Oct 2023 for DS), potentially giving GPT access to more up-to-date foundational science content and exam-relevant information. Our results suggest that GPT has a more extensive medical knowledge base than DS, as reflected in its stronger performance on Step 1 questions. However, the performance gap between the two models was narrower on Step 2 questions, which emphasize clinical reasoning, suggesting that their applied reasoning capabilities may be more comparable. Therefore, students should prioritize the use of GPT for Step 1 examination preparation.

GPT demonstrated significantly higher accuracy across all three difficulty levels, but the significance did not persist for easy questions after stratifying by examination type. Accuracy of both models declined as question difficulty increased. The performance gap between GPT and DS increased as question difficulty increased for both examination types. These findings align with a prior study indicating an inverse correlation between question difficulty and performance of GPT [11]. While both models struggle with more difficult questions, the results show that GPT remains more reliable than DS for difficult questions on either examination. Our interpretation is that students may use either AI model for easy-level questions. For difficult questions, caution is advised when using either model, with GPT recommended over DS.

Specific topic performance gaps between the two AI models were inconsistent across examination types. None of the statistical significances for topics in Step 1 questions were consistent for the same topic in Step 2 questions. Given that the topic-based performance gap is more prominent in Step 1 than Step 2 questions, it is reasonable to imply that the difference is driven more by question style than by topic. It is possible that GPT performs better than DS in mechanism/knowledge questions seen more commonly in the Step 1 examination. Nevertheless, students should still reference our results when selecting AI models for specific medical topics (reference Figure 3) because the difference in accuracy can be a compounded effect between examination type and medical topic. GPT should be prioritized for most topics except “human development” and “Step 2 blood and lymph,” where DS may be equally or more suitable.

Practical implications for medical students

Our findings recommend that students choose GPT-4o for USMLE study over DS, regardless of question difficulty, topic, or examination. However, DS remains a viable alternative in settings where GPT is not accessible due to cost or censorship restrictions. Currently, GPT-4o requires a paid Plus or Pro subscription for consistent availability, which costs at least 20 USD per month. For users without the upgrade, GPT does not specify which models are being used to generate questions, such as 4o mini, o1, o3, o3mini, etc. No studies have evaluated the performance of these free models on USMLE-style questions, though a prior study [3] showed GPT3.5 had an overall accuracy of only 46.22% for both Step 1 and Step 2. Therefore, the reliability of free-tier GPT models remains uncertain. In contrast, DS is free and provides a consistent experience for all users, making it a potentially more reliable choice for those without access to GPT-4o. Despite its overall inferiority to GPT, DS remains a viable study tool for specific students, but caution is advised when using it for intermediate and hard Step 1 questions.

Study limitations

Our study has several limitations. Our question sets did not include questions with tables or images. While GPT-4o is capable of processing graphs and tables, DS currently lacks this functionality. As a result, results from image-focused topics, such as dermatology and musculoskeletal topics, may be subject to selection bias. Additionally, all questions in this study were sourced from one question bank. Although AMBOSS is a highly respected and utilized USMLE study question bank, caution should be taken when generalizing results to all USMLE questions. Future studies should assess AI performance across multiple question bank sources and incorporate image-based questions to provide a more comprehensive evaluation.

## Conclusions

Our findings underscore GPT’s superior accuracy in answering USMLE-style questions, particularly in intermediate and hard categories. While both AI models demonstrated strong performance compared to human learners, GPT's consistently higher accuracy across most medical topics makes it the preferred tool for AI-assisted medical education at this time. Importantly, the variability in AI performance across different medical systems suggests that students can strategically use AI models based on their strengths in specific subject areas. As AI continues to integrate into medical education, further research is needed to refine these models and ensure their reliability across all medical domains.
